# The Application of Human–Computer Interaction Technology Fused With Artificial Intelligence in Sports Moving Target Detection Education for College Athlete

**DOI:** 10.3389/fpsyg.2021.677590

**Published:** 2021-07-22

**Authors:** Jie Liu, Le Wang, Hang Zhou

**Affiliations:** ^1^Sports Training and Health Care, Zhoukou Normal University, Zhoukou, China; ^2^Sports Training, Zhongyuan University of Technology, Zhengzhou, China; ^3^Computer Science and Technology, Zhoukou Normal University, Zhoukou, China

**Keywords:** artificial intelligence, framework of coaching system, motion capture technology, human-computer interaction, sports training

## Abstract

The purposes are to digitalize and intellectualize current professional sports training and enrich the application scenarios of motion capture technology of moving targets based on artificial intelligence (AI) and human–computer interaction (HCI) in sports training. From an educational psychology perspective, sport techniques are a cognitive ability of sports, and a tacit knowledge. However, sports technology, language, image, and other methods play an auxiliary role in sports training. Here, a General Framework of Knowledge-Based Coaching System (KBCS) is proposed using the HCI technology and sports knowledge to accomplish autonomous and intelligent sports training. Then, the KBCS is applied to table tennis training. The athletic performance is evaluated quantitatively through the calculation of the sports features, motion recognition, and the hitting stage division in table tennis. Results demonstrate that the speed calculated by the position after mosaicking has better continuity after the initial frame of the unmarked segment is compared with the end frame of the market segment. The typical serve and return trajectories in three serving modes of slight-spin, top-spin, and back-spin, as well as the trajectories of common services and return errors, are obtained through the judgment of the serving and receiving of table tennis. Comparison results prove that the serving accuracy of slight-spin and back-spin is better than that of top-spin, and a lower serve speed has higher accuracy. Experimental results show that the level distribution of the three participants calculated by the system is consistent with the actual situation in terms of the quality of the ball returned and the standard of the motion, proving that the proposed KBCS and algorithm are useful in a small sample, thereby further improving the accuracy of pose restoration of athletes in sports training.

## Introduction

Nowadays, sports have been vigorously promoted by all social spheres and are closely related to the daily life and social activities of people. Traditional sports training and evaluation methods almost solely depend on coaches for targeted training, which have shown some disadvantages. First, with the surging sports population, professional coaches are severely out of demand, so some fitness people cannot get in-depth guidance. Second, the decisions of coaches are not always reliable, which are mostly based on experiences and lack of scientificity. Given the above situation, the intelligent reform of sports training has become a hot topic of research. Particularly, in various high-profile professional sports events, the battle for data is intensifying, and big data has become another arena outside the court (Shih, 2017). In addition, digital technology is increasingly integrated into mass sports. Professional sport has entered the era of digital competition ahead of time, and the era of solely relying on the talent of athletes and the experience of coaches has gradually faded out (Mazzeo et al., 2018). Big data services, including the collection and analysis of scores, health data, and game data of athletes, are becoming an indispensable means for professional clubs to discover their strengths and weaknesses to win (Rein and Memmert, 2016). For example, in the National Basketball Association (NBA) of the United States, all NBA courts are equipped with the Sports VU Player Tracking System, including cameras and special software (Guan et al., 2019), which provides continuous data streams and innovative statistics to analyze the motions of all athletes, including speed, distance, emergency stop, steering, acceleration, the dynamic distance between athletes, motion patterns of the joints of each athlete, and ball control. A series of in-field data, such as points, rebounds, assists, blocks, steals, turnovers, and fouls, have been analyzed in detail to provide suggestions for each athlete to break through their limitations and take advantage of others (Patel et al., 2020). In China, digital sports are in a stage of leapfrog development; particularly, digitalization has become the key to the quality control of physical education (PE) (Pan, 2019). Athletic abilities include professional skills, professional intelligence, physical fitness, and psychological quality. Especially, for excellent athletes whose intelligence and professional skills have reached a fairly high level, the impact of physical fitness on their sports career may be crucial (Liu and Zhu, 2020). According to the situation of athletes, scientific and effective physical exercises are made, which can not only help athletes approach the limit of sports ability, improve the effect of sports training, but also effectively prevent sports injury. Therefore, modern competitive sports put forward higher requirements for physical exercise (Di Zhang et al., 2020). Physical exercise is a very complex system, whose reasonable and controllable engineering depends on digital scientific monitoring (Lee and Han, 2019). The *2018 China Fitness Trends* and *2018 Global Fitness Trends* jointly released by the Shanghai Institute of Sport and the American Society of Sports Medicine in Shanghai suggest that wearable devices for data collection and analysis rank the second, including system tracking equipment, smartwatches, heart rate monitoring equipment, and Global Positioning System (GPS) equipment (Luo et al., 2019).

Educational psychology is a branch of psychology based on materialist dialectics and diverse research methods, which extracts the essence of different theoretical schools to systematically explore the psychological state of people in sports from different angles and levels (Li and Xu, 2018). Educational psychology plays an important role in PE teaching. The relevant concepts of educational psychology can be applied to the research of psychological treatment, psychological relief, PE teaching, and training of students (Van den Berghe et al., 2014). In the present stage of PE teaching, the concept of educational psychology should be combined with practice to fully understand the individual characteristics of middle school students, appropriately adjust the application of sports psychology, and improve the relevant teaching methods (Vasconcellos et al., 2020). More importantly, PE should be taught according to the aptitude of students, namely, to design a teaching and training plan suitable for the students according to their specific conditions. To achieve this goal, in addition to the investment and attention of teachers, an advanced PE teaching system should be developed to assist teaching (Haerens et al., 2015). The function of the coach includes two parts: to observe the training content and the performance of athletes and to guide the athletes in training (Namli and Demir, 2020). The digital sports training system can record the training process through motion capture technology, analyze the training effect through accurate modeling and calculation, and give appropriate guidance and feedback (Kroon and Eriksson, 2019). The original motion information can be more accurately and transparently obtained through the motion capture device than a coach, which is also easier to record, save, and analyze in the future. Automatic evaluation and training based on this information can reduce the dependence on the experience of the coach (Wunderlich and Memmert, 2020). It is necessary to explore the motion capture technology and digital sports training based on motion capture to achieve the digitization and automation of sports training (Su, 2020). A comprehensive physical fitness test comes first in the initial training stage. The test results are analyzed and evaluated so that the coach can master the physical features of each athlete and then periodically formulate a feasible phase physical training plan (Yuan et al., 2020). Following the physical training system, coaches can master the indicators of training of athletes, make targeted training plans, and further improve the training effect (Brandao et al., 2019).

Here, the concept of teaching students following their aptitude in educational psychology research is integrated into PE teaching, and a training framework for autonomous sports training guidance system of athletes is proposed. The most mature table tennis in Chinese sports activities is selected for research, and the training situation is systematically analyzed for college athletes in table tennis. Consequently, a general framework of Knowledge-Based Coaching System (KBCS) is proposed for autonomous sports training. It uses human–computer interaction (HCI) motion capture technology in sports training to monitor sports data and introduces a digital training framework to implement personalized feedback based on motion capture data. The four principal modules, namely, domain knowledge, athletes model, sports evaluation, and controller, are utilized as clues to investigate the current research on sports auxiliary training, pointing out that an integration of the above four modules and a general and complete digital sports training framework is lacking. Hence, a table tennis coaching system based on hybrid motion capture technology is built and verified. The experiment invites table tennis athletes of different levels to verify the accuracy of the system data measurement and the correctness of the framework process, laying a foundation for further research on the highly precise and interactive sports coaching system. The selected athletes of different grades are those whose average sports ability is at the upper, middle, and lower levels, respectively. The division is mainly based on the overall training performance of the athletes, and it is decided by the coach through joint discussion and evaluation.

## Methods

### Motion Capture Technology in Sports Training

Motion capture is a technology that can digitally record the human poses (position and pose) or objects by collecting information, such as inertial data of human motion, markers, or moving images, after algorithm processing (Choe et al., 2019; Merino et al., 2019). Currently, the mainstream motion capture technology includes two categories: One is optical capture, and the other is inertial capture; in a capture effect perspective, the accuracy of inertial capture is not as good as that of optical capture (Yu et al., 2019; Menolotto et al., 2020). The performance comparison results of different motion capture technologies are shown in Table 1.

**Table 1:** Performance comparison of different motion capture technologies.

**Performance indicator**	**Inertial**	**Optical non-calibration**	**Optical calibration (Active)**	**Optical calibration (Passive)**
Accuracy	High	High	High	High
Computational efficiency	High	Low	Low	Low
Range of motion	Large	Small	General	General
Multi-Target motion capture	General	Low	General	High
Environmental constraints	Sensor interference	Heat source interference	Strong light source interference	Sunlight interference
Cost	Low	Low	Medium	Medium

**Figure 1:**
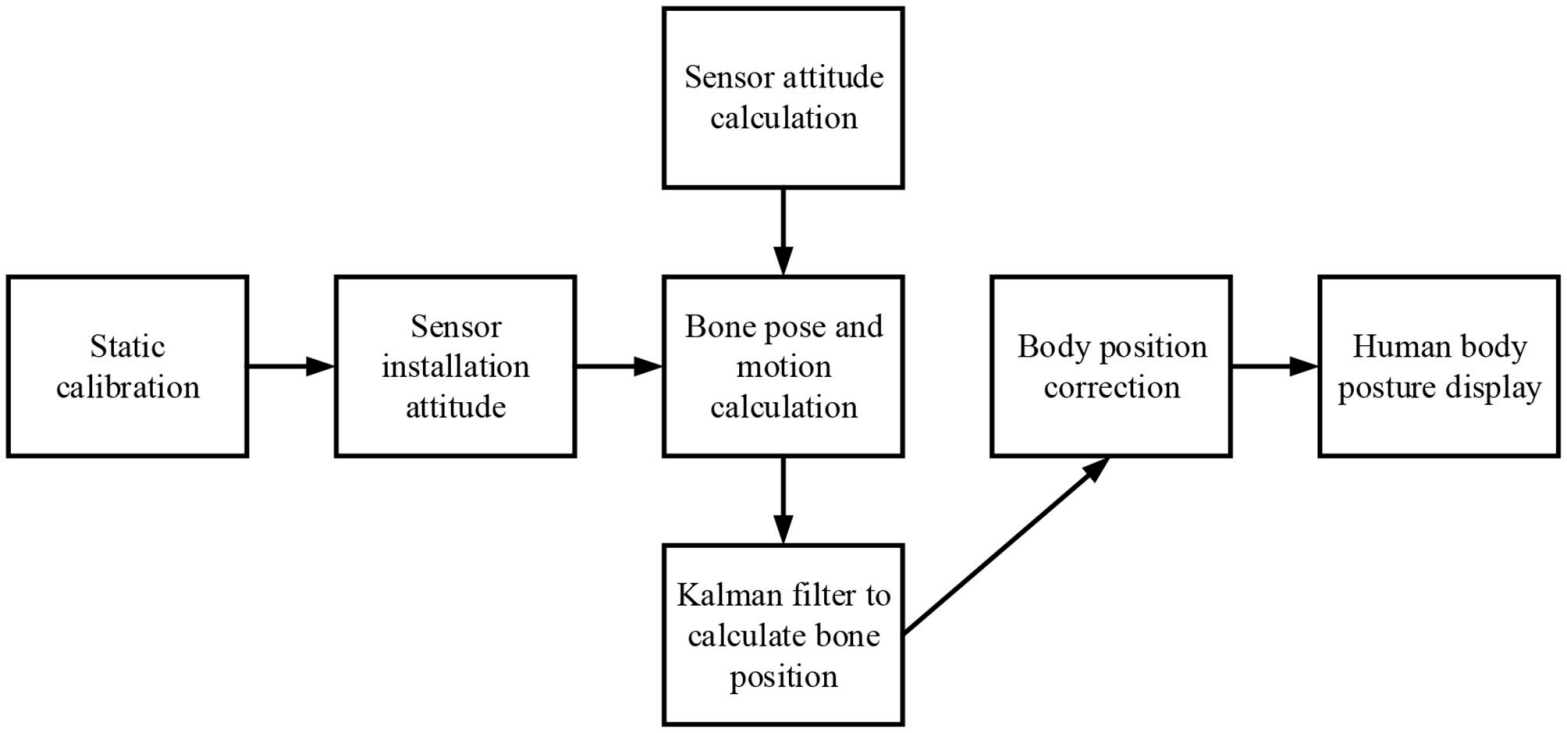
The fundamental process framework of inertial motion capture technology.

Bodenheimer et al. focused on the software level, assumed the human skeleton as an articulated steel structure, used offline optimization methods to determine the bone length, and applied an inverse kinematic chain to generate motion trajectory (Bodenheimer et al., 2002). However, the problem of multiple solutions was not well-solved, and the restoration accuracy was limited. Fontaine was committed to building the motion capture device into a new generation of HCI interface, emphasizing more on the acquisition and calculation of raw data from the hardware level. Also, the selection and wearing of sensors were considered, and the errors that might be involved in the entire calculation process were explained (Fontaine et al., 2002). Therefore, the fundamental research framework of inertial motion capture and the principal problems to be solved are determined, as shown in Figure 1.

### Construction of a Digital Coaching Framework Based on Educational Psychology

Digital sports training monitors the poses of athletes and changes in equipment with motion capture devices and other sensors in sports training. Then, based on the monitored information, the last sports performance of athletes can be evaluated to provide them with new training content or training instructions accordingly (Appelbaum and Erickson, 2018). Specifically, it includes the digitization of sports knowledge expression, the digitization of sports performance records, and the digitization of training content/instruction generation. The digitization of sports knowledge expression is the explicit expression of the subjective experience of the coach as words or numbers (Fister et al., 2015). The digitization of sports performance records captures the original sports information related to the indicator calculation according to the skill indicators tested by the sports events. Different sports have different skill requirements for athletes, and the original information content and accuracy requirements that need to be captured are different (Wang et al., 2018). Human motion capture includes the center of gravity position, exercise time, exercise frequency, the distance between limbs, and joint angles. Information monitoring of sports equipment includes ball trajectory and racket acceleration in small ball sports, such as table tennis and tennis, and boat hull acceleration in rowing sports (Kari and Karhulahti, 2016).

Reference library *T* stores the various skill indicator scores of high-level athletes or the standards of a particular level for athletes to choose from. Ability level and practice content are coupled. Under the premise of ensuring the credibility of the correlation between each practice group and each skill level, each skill level should be refined as much as possible to increase the distinction between different practice groups. The exercise library *X* covers all exercises involved in sports. All practice content can be divided into two levels: group and difficulty. Standard tests of different difficulty levels are also part of the practice library; however, the content that differs from the interactive practice is listed separately.

The instruction library *C* stores all the feedback instructions involved in the training process, and different instructions are independent of each other. The command generation in the training process is also a process of gradual optimization. The similarity comparison based on sports performance reduces the search space and improves the convergence efficiency of the optimal feedback command.

**Figure 2:**
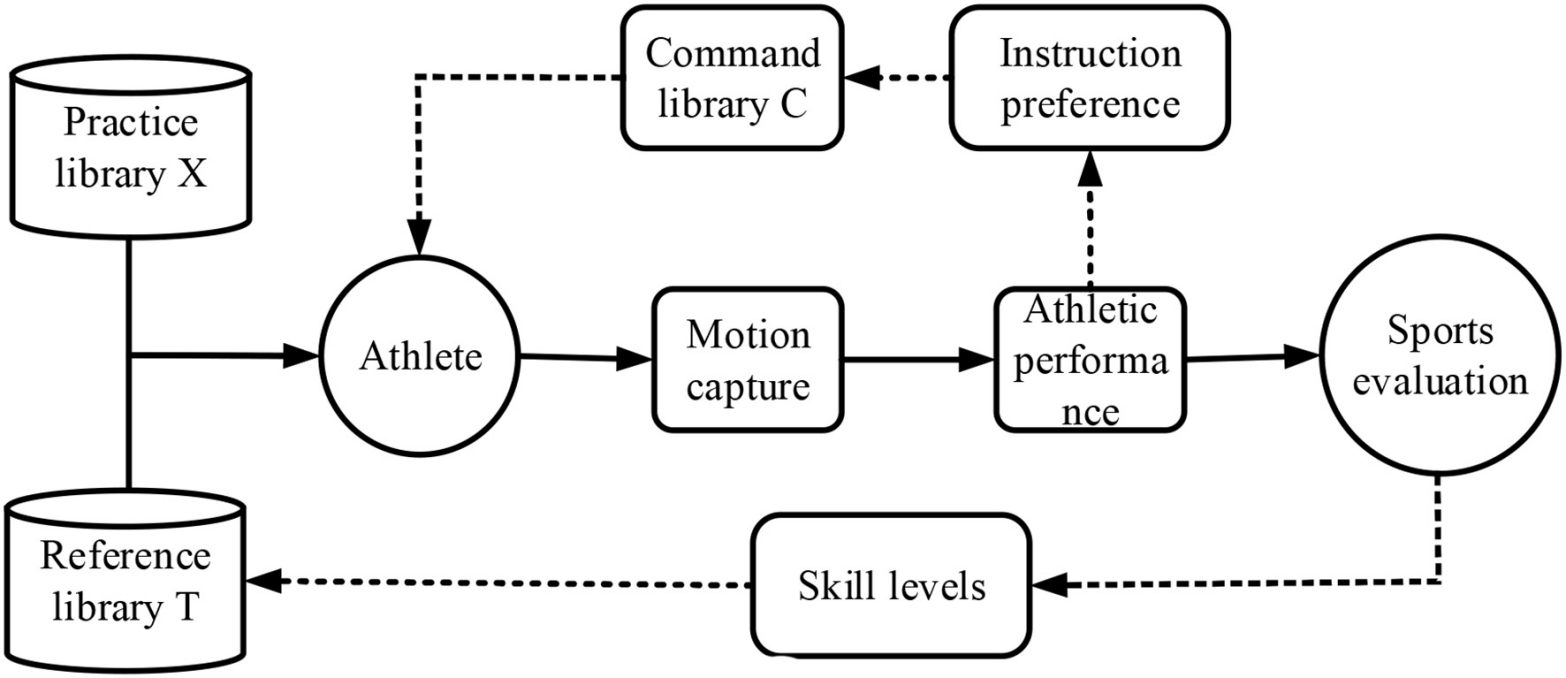
A schematic diagram of KBCS.

The General Framework of KBCS is illustrated in Figure 2.

### Motion Capture Calculation in Sports Training

Specifically, the evaluation method of a sport expresses subjective experience and evaluation principles of coaches as objective and measurable sports features by communicating with professional coaches (Hüttermann et al., 2018; Tyson et al., 2018). According to the sequence of program processing and the degree of data abstraction, motion evaluation can be divided into three levels: motion capture, feature extraction, and motion evaluation.

A large amount of original sports data *m* can be obtained through motion capture technology. Reliable sports evaluation not only depends on the monitoring of movement data of athletes but also requires sports equipment data as support. The original motion capture data include but are not limited to the bone pose of athletes, acceleration, ground contact, and trajectory of sports equipment. The equation is expressed as:

(1) $$\begin{array}{r} m={\left(x,c,t\right)}^{3} \end{array}$$

Feature extraction is based on the original motion capture data. After the motion segmentation and motion analysis are combined with the features of the sports item itself, the process *p* is calculated to obtain the instant performance (motion feature). The extracted motion features can generally be classified into three categories: time correlation, kinematics, and dynamics. Table tennis is taken as an example. The three-dimensional trajectory of the ball and racket is the original motion capture data, and the acceleration of the racket and the flight time of the ball before each round are the motion features. The equation is expressed as:

(2) $$\begin{array}{r} p=e\left(x,c,t\right) \end{array}$$

Sports evaluation is the process of calculating skill indicator scores from instant performance. Compared with sports features, the definition of skill indicators is more abstract, and correspondingly, its calculations are more complicated. Because a certain exercise content is not strongly related to all skill indicators, only the value ŷ of the related skill indicator can be calculated based on the instant performance under a single exercise content.

The relational equation of the three levels can be expressed as:

(3) $$\begin{array}{r} ŷ=h\left(p\right)=h\left(e\left(m\right)\right) \end{array}$$

The function of the controller in KBCS includes updating the skill level of athletes after the exercise evaluation, providing new exercise content after the current exercise content is completed, and providing feedback instructions when the action response of the current exercise content is wrong.

The equation for skill-level update is:

(4) $$\begin{array}{r} {y^{\prime }}_{tra}=y_{tra}⊕ŷ \end{array}$$

In (4), *y*_*tra*_ represents the current skill level of athletes, and ⊕ represents the data fusion of the two common indicator values.

The equation for selecting exercise content is:

(5) $$x^{*}=\;arg\;max(relu\;(y_{ref}\;-\;y_{tra}\;\mu ((xi))$$

where *y*_*ref*_ is the reference value, and the function *relu* indicates that the negative value in the vector is taken to zero, while the other elements remain unchanged, and ▪ indicates the inner product of the two vectors.

The equation for selecting the feedback command is:

(6) $$\begin{array}{r} pr_{tra}\left(c|x,p\right)=\frac{d\left(\left\{x_{i},p_{i}\right\},\left\{x,p\right\}\right)}{\overset{v}{\underset{j=1}{\sum }}d\left(\left\{x_{j},p_{j}\right\},\left\{x,p\right\}\right)} \end{array}$$

where *pr*_*tra*_(*c*|*x, p*) represents the preference of athletes when their real-time performance on exercise content *x* is *p*, and each instruction is selected for feedback. The preference of each instruction ranges from 0 to 1. The larger the value, the higher the preference.

### The HCI Sports Training Based on Educational Psychology

Athletes need to select a learning objective from the reference library. Afterward, they select the standard test of particular difficulty. Their skill levels and the difficult progress of all exercises are initialized. According to the skill levels of athletes, reference target levels, content, and indicator relevance, the system can calculate the optimal exercise group and exercise difficulty suitable for athletes (Yang et al., 2019). If the real-time performance of athletes under a given exercise content reaches expectations, the system will select new exercise content for athletes after updating the difficulty progress and related skill indicators in the exercise group. Otherwise, the system will calculate the similarity between the current real-time performance and the existing performance in the command library, select the command with the highest similarity (or preference) to feedback to athletes, and update the command preference according to the feedback effect. The termination condition of interactive training is that the skill levels of athletes are not much different from the reference skill levels.

**Figure 3:**
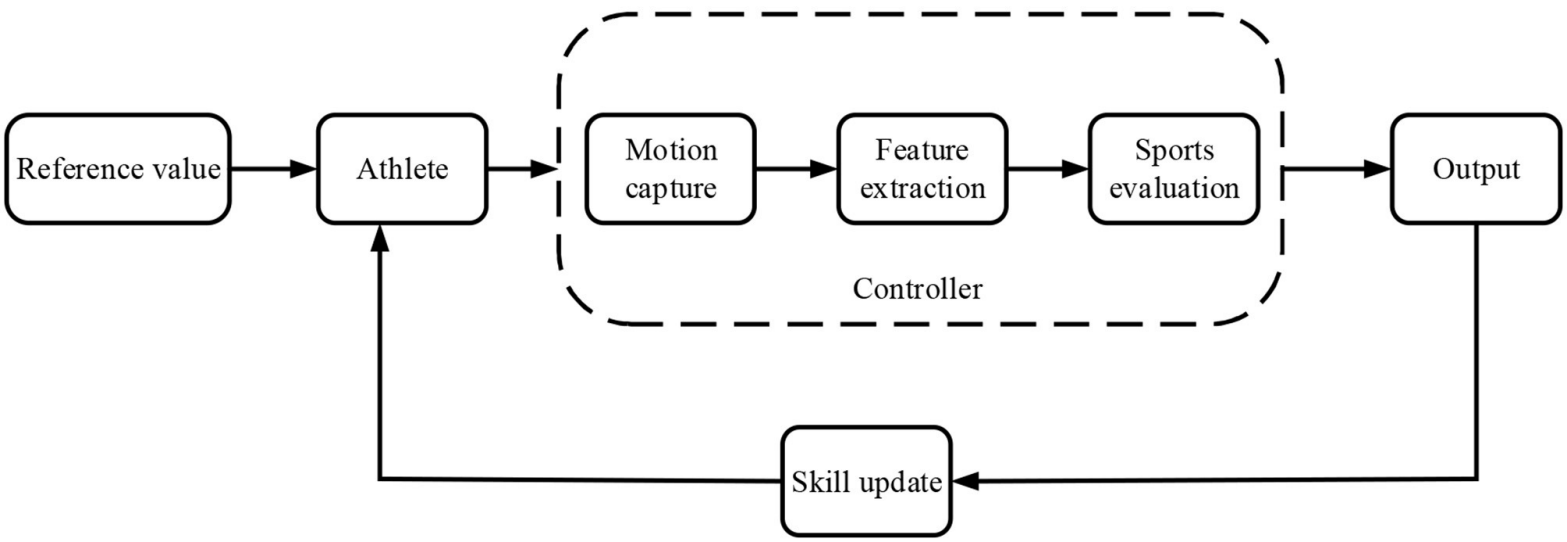
The HCI training process of the sports coaching system.

If the athletes feel that the system updates their skill levels incorrectly or causes a change in the skill levels during self-practice, they can calibrate various skill indicator values through standard tests. Then, the system will continue interactive training based on the updated skill levels. The flowchart of the entire training process is shown in Figure 3.

### Application of HCI Coaching System in Table Tennis

**Figure 4:**
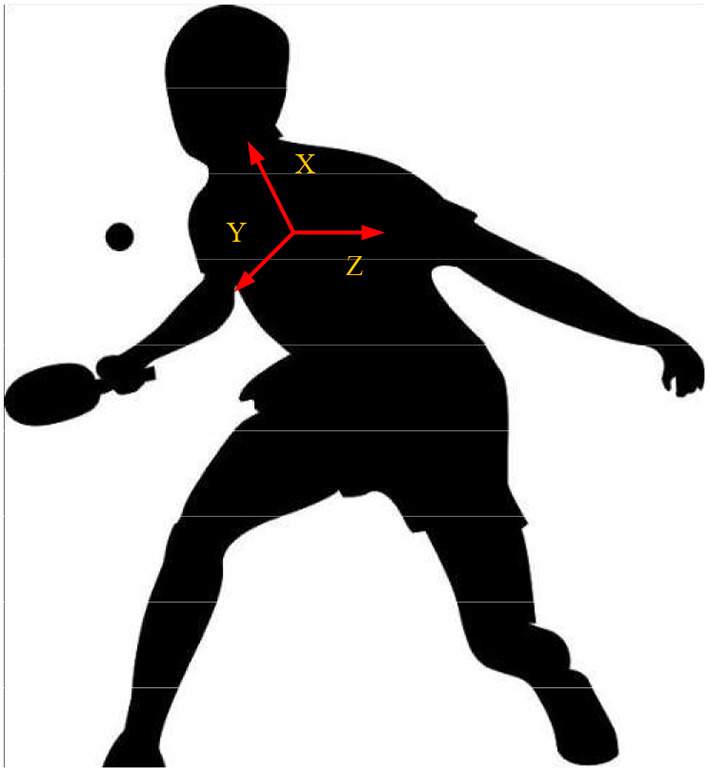
Axis points of human bones.

The proposed KBCS is applied to the field of table tennis. The implementation of KBCS mainly considers two aspects: implementation difficulties and error handling, that is, clear practice content, instant performance, the meaning of each element in the skill indicator, and distance calculation. In addition, KBCS deals with the serve execution error and motion capture measurement error to make the errors within the acceptable range. The original inertial motion capture system is improved from two aspects: contact judgment and pose correction based on contact position constraints further to improve the accuracy of the pose restoration of athletes. The inertial system can directly output the position, velocity, acceleration, angular velocity, and pose of the main bones of the human body. Among them, the bone pose has the highest credibility, and the estimation of the bone position has a large deviation. The binding relationship between the racket and the athletes when hitting the ball can improve the accuracy of human body position capture in the experiment, and the position of the racket calculated by the optical system can correct the bone position of the human body for the first time. Among human features, position-related features can be directly calculated based on the corrected bone position, such as the distance between feet. The calculation of joint angles can refer to the method provided by the International Society for Biomechanics (ISB): The anatomical pose of the human body is defined as the zero position, the one closest to the head of the two adjacent bones is defined as the proximal limb, and the three directions of “up-front-left” in the zero pose correspond to the directions of the “X-Y-Z” coordinate axes of each bone (Chen et al., 2020), as shown in Figure 4.

The external rotation angle, flexion angle, and abduction angle of each joint can be obtained from the pose of the distal limb relative to the proximal limb. The calculation is:

(7) $$\left[\theta_{\alpha },\theta_{\beta },\theta_{\gamma }]=T_{rxzy}{(}_{prox}^{dist}q\right)$$

where *T*_*rxzy*_ indicates that the pose rotation sequence is its X-Z-Y coordinate axes, and the result is a three-dimensional vector, corresponding to the external rotation angle, flexion angle, and abduction angle in order; ${\;}_{prox}^{dist}q$ indicates that the pose of the distal bone relative to the proximal bone is in quaternion form.

### Bone Position and Pose Correction in the Experiment

The lower limbs of the human body are specifically studied to explore a method of correcting the pose of the human body based on an optimized framework. The lower limbs of the human body are treated as two kinematic bone chains, and the pelvic position is the common starting point of the two kinematic chains. If only the optimized pose can satisfy the contact position constraint, there are multiple solutions for the pose adjustment of the two kinematic chains. Thus, the following assumptions are made to reduce the solution space and increase the solution speed: The position deviation of the pelvis before and after optimization and the position deviation of the two kinematic chain nodes (thighs and calves) should be as small as possible. The optimized pose of each frame in the animation should not be too different from the optimized pose of the previous frame to avoid visually causing the “jitter” and distortion of the characters in the animation.

Based on the above assumptions, the optimized equations are as follows.

(8) $$\underset{p_{hip,}R_{ij}}{min}{\left(p_{hip}-p_{hip0}\right)}^{2}+{\sum_{i=1}^{m}\sum_{j=1}^{n_{i}}\left(R_{ij}r_{ij}-R_{{\left(ij\right)}_{0}}r_{ij}\right)}^{2}$$

(9) $$\begin{array}{r} p_{hip0}=\alpha p_{hip}^{last-frame}+\left(1-\alpha \right)p_{hip}^{measure} \end{array}$$

(10) $$\begin{array}{r} R_{{\left(ij\right)}_{0}}=\beta R_{ij}^{last}+\left(1-\beta \right)R_{ij}^{measure} \end{array}$$

(11) $$\begin{array}{r} E_{i}=P_{hip}\;+\;\overset{n_{i}}{\underset{j=1}{\sum }}R_{ij}r_{ij} \end{array}$$

In the above four equations, *p*_*hip*_ represents the estimated position of the pelvis, *p*_*hip*0_ represents the initial position of the pelvis, denotes the number of kinematic chains, *n*_*i*_ denotes the number of bones corresponding to the *i*-th kinematic chain, *R*_*ij*_ stands for the estimated pose of the *j*-th bone corresponding to the *i*-th kinematic chain, stands for the initial pose of the -th bone corresponding to the *i*-th kinematic chain, *r*_*ij*_ refers to the length vector of the *j*-th bone corresponding to the *i*-th kinematic chain in the upright attitude, and *E*_*i*_ refers to the true value of the end position of the *i*-th kinematic chain.

### Experimental Methods and Environment

Changes in the abilities of athletes after using the system for a long time are not considered to shorten the experimental verification cycle. Only the one-way open-loop process of serving the ball → receiving the ball → motion evaluation → feedback command is considered. In other words, under the same exercise difficulty test, if the exercise evaluations of different athletes calculated by the system are consistent with the actual situation, the system built and the calculation rules adopted are considered valid within the range represented by the sample. Besides, under the premise that the level of athletes can be distinguished, the service of table tennis is limited to three types: light-spin, top-spin, and back-spin to reduce the time required for the experiment of a single athlete and enrich the number of samples. Other serving modes are not considered. According to the experimental requirements, an automatic serve machine, the inertial motion capture system, and the optical motion capture system are selected, and the reflective treatment of table tennis and racket is explained. Among them, the automatic serve machine is Shuangyu 1040 automatic serve machine. The running environment of the inertial capture system and optical capture system is the Window10 system. During the experiment, the computer CPU model is Intel i7-9700k, and the memory is 8G. The optical motion capture system is the Nokov, which can capture the key points of the trajectory at a higher frame rate. At the same time, the system has good stability and is suitable for the experimental requirements. The Legacy system is selected for inertial motion capture, which can output the posture of the main human bones more accurately. Meanwhile, the sensor of the Legacy system has a long life and can meet the application for a long time.

The experiment requires three participating athletes to try three different levels of service (slight-spin, top-spin, and back-spin). Specifically, under a particular difficulty, the automatic serve machine is set as a fixed-point serve to the forehand position of athletes to ensure that the athletes of the lowest level can effectively receive the ball in the minimum difficulty. Athletes need to return 7 to 10 balls to the diagonal, centerline edge, and edge corners of the opposite table, under the condition that priority is given to ensuring that the returned ball can fall on the table.

At the beginning and end of the experiment, the participants hold a reflective racket and knock on the desktop, causing sudden acceleration on the rigid body of the racket in the optical system (or the reflective point on the racket) and the sensor on the holding hand in the inertial system. In a single round, the trajectory of the ball and the action of the participant are consistent in time, and the time starting point of the two systems has been synchronized. Therefore, the inertial data of human body motion in the corresponding period can be located by detecting the time when a marked table tennis ball appears in the optical system. After the above data processing, the typical service and return trajectories under three serving modes of slight-spin, top-spin, and back-spin can be obtained.

## Research Results

### Pre-processing Results of Optical Motion Capture Data of the Table Tennis Coaching System

**Figure 5:**
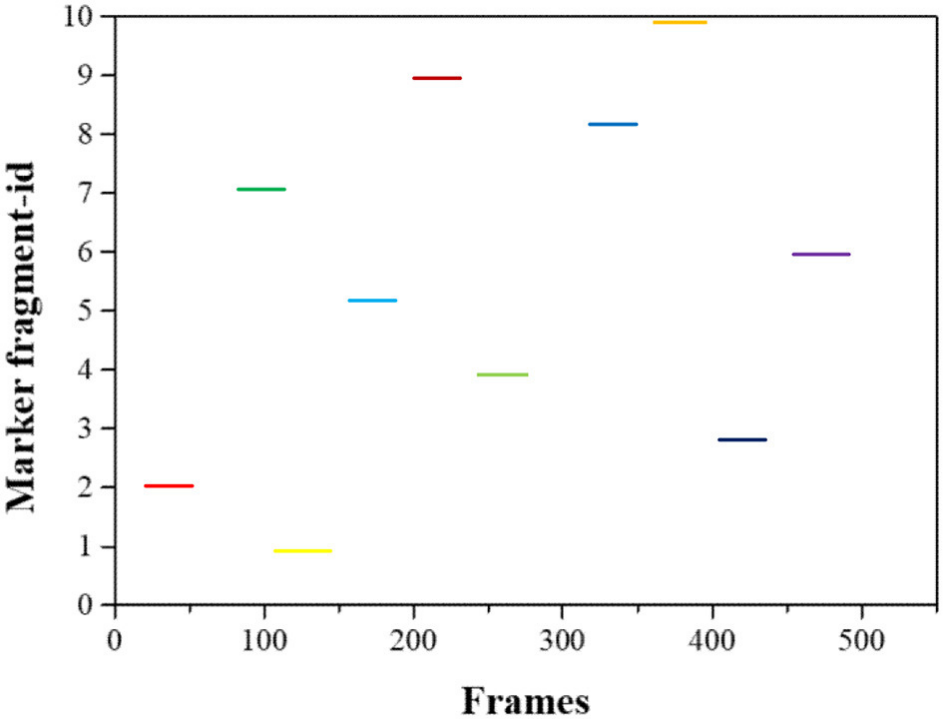
Marked segment before pre-processing.

**Figure 6:**
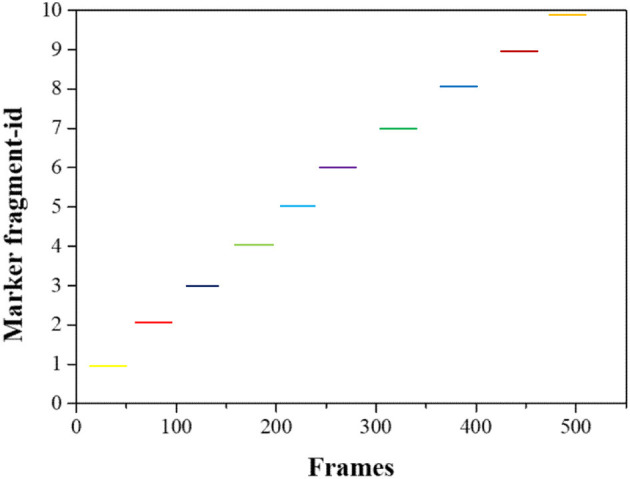
Marked segment after pre-processing.

The initial frame of the unmarked segment and the end frame of the marked segment are compared using the temporal and spatial features of the table tennis flight. If the time difference between the two frames of data is very small, and the speed calculated by the position has good continuity after mosaicking, it is considered that the two segments correspond to the flight trajectory of the same table tennis, and they are mosaicked. Changes in the data processing effect are shown in Figures 5, 6, which means that within the time range of 500 frames, the unmarked data segment is cut to the corresponding market segment according to the continuity of time and space.

### Results of Table Tennis Serve Detection Judgment

**Figure 7:**
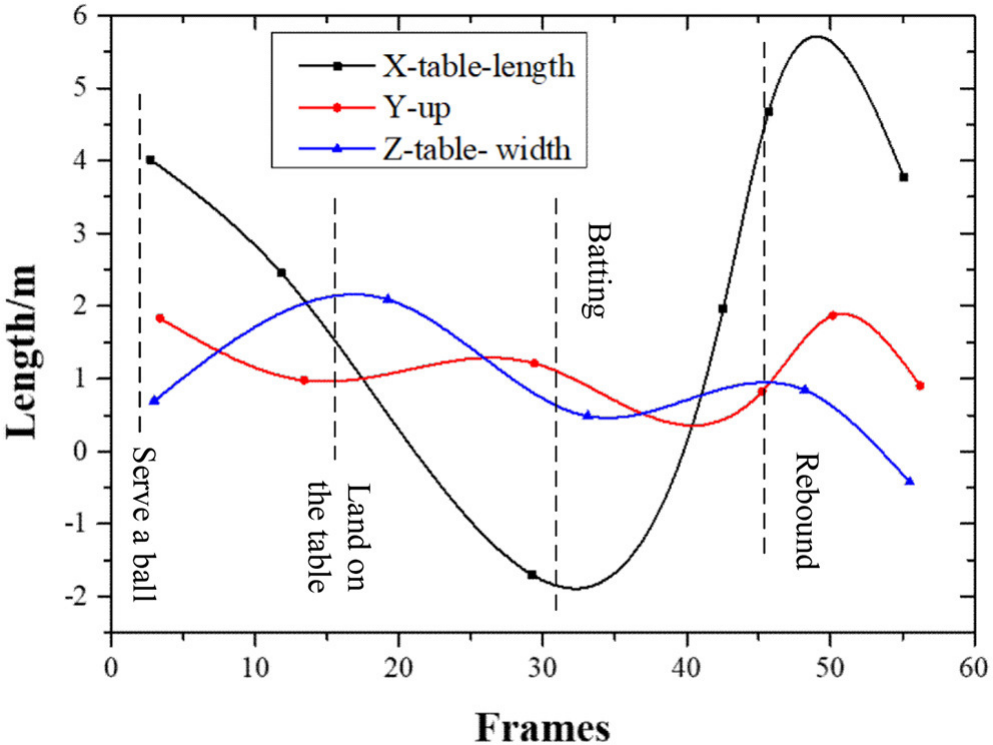
A single-round table tennis trajectory corresponding to slight-spin serve.

The single-round table tennis trajectory corresponding to slight-spin serve is shown in Figure 7.

The X-axis of the coordinate system in Figure 7 is along the long side of the table, pointing from the athletes to the direction of the automatic serve machine. The Y-axis is vertically upward, and the Z-axis points to the short side of the table according to the right-hand rule. Four key points in a single round, such as a service, missed service, hitting, and returning to rebound, have been marked in the corresponding positions in Figure 7.

**Figure 8:**
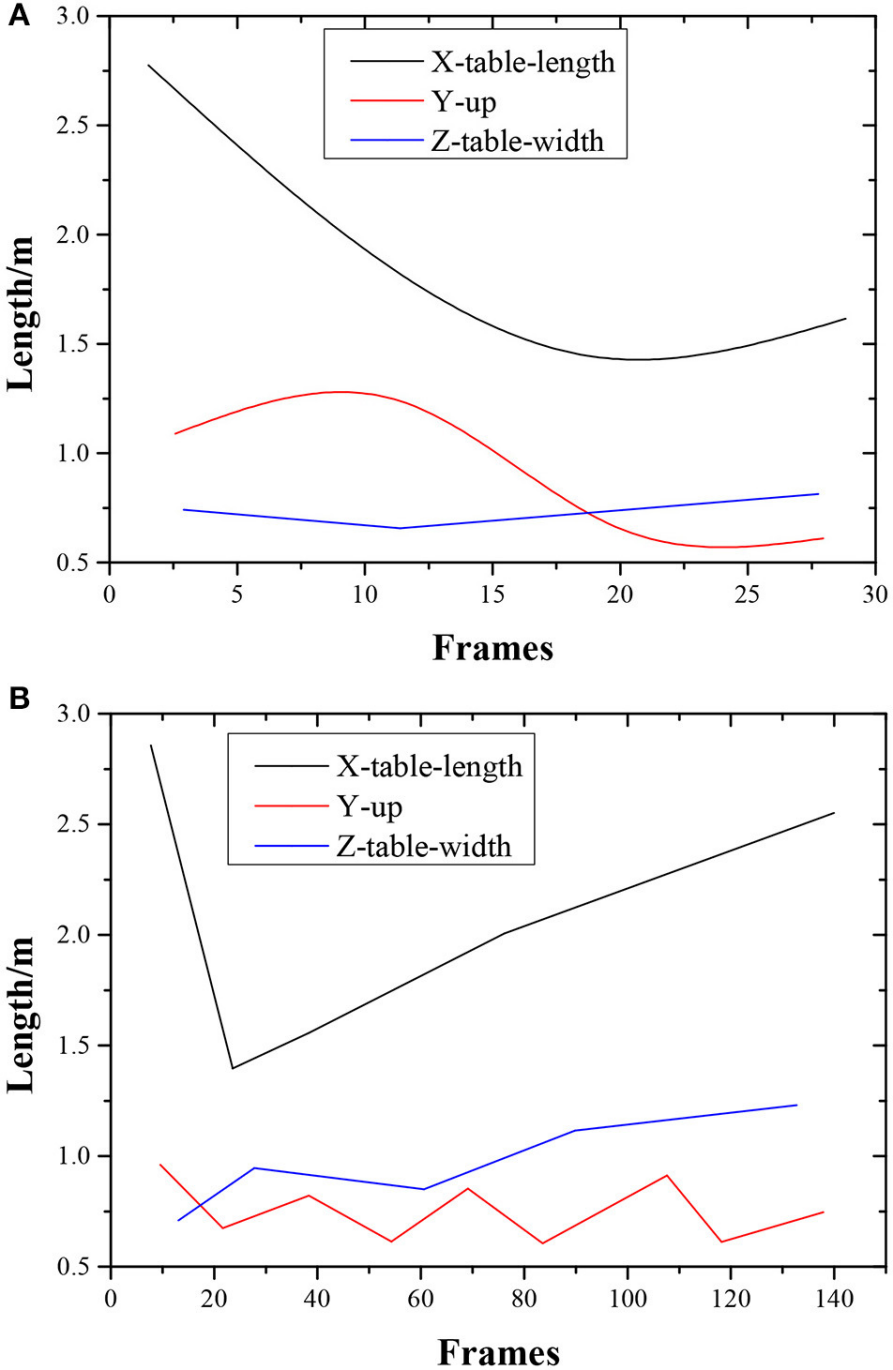
**(A)** Table tennis trajectory of the serve touching the net. **(B)** Table tennis trajectory of the missed serve.

The common serve errors in the experiment are as follows. (1) The service is blocked by the net, or (2) the first drop point is on the side of the automatic serve machine, as shown in Figure 8.

When the serve touches the net, the X-axis (along the long side of the table) direction of the trajectory only reaches half the length of the table. When a missed serve occurs, the ball rebound prematurely in the Y-axis (vertical upwards) direction of the trajectory. The sag appears in the trajectory on the X-axis is because the ball rolls slowly to the side of the tee after bounced to the net.

### Results of Table Tennis Receiving Detection Judgment

**Figure 9:**
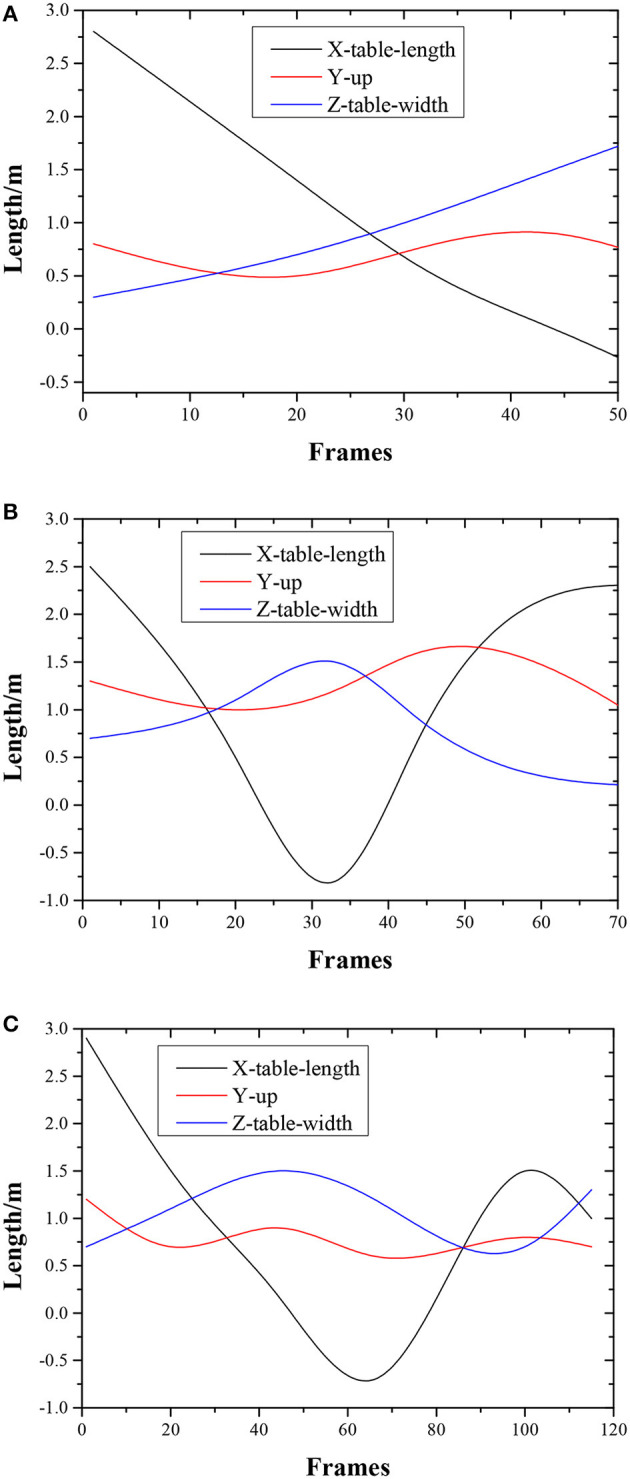
**(A)** Table tennis trajectory of missed receiving. **(B)** Table tennis trajectory of receiving the ball out of the table. **(C)** Table tennis trajectory of receiving the ball touching the net.

There are more common errors in ball receiving, including the missed receiving, receiving the ball out of the table, and receiving the ball touching the net. The table tennis trajectories in the above three situations are shown in Figure 9.

Figure 9A shows that athletes fail to receive the ball, and the corresponding X-axis coordinate drops from the initial value (on the server side) to zero (on the athlete side). In the meantime, the Y-axis maintains a stable approximate parabolic curve after the first rebound from the table touch. Figure 9B shows the situation where the ball is returned but does not touch the table of the opponent because the Y-axis direction of the trajectory does not rebound after the ball is hit. Figure 9C represents the situation of the table tennis trajectory where the received ball touches the net.

### Results of Serving Quality Analysis

**Figure 10:**
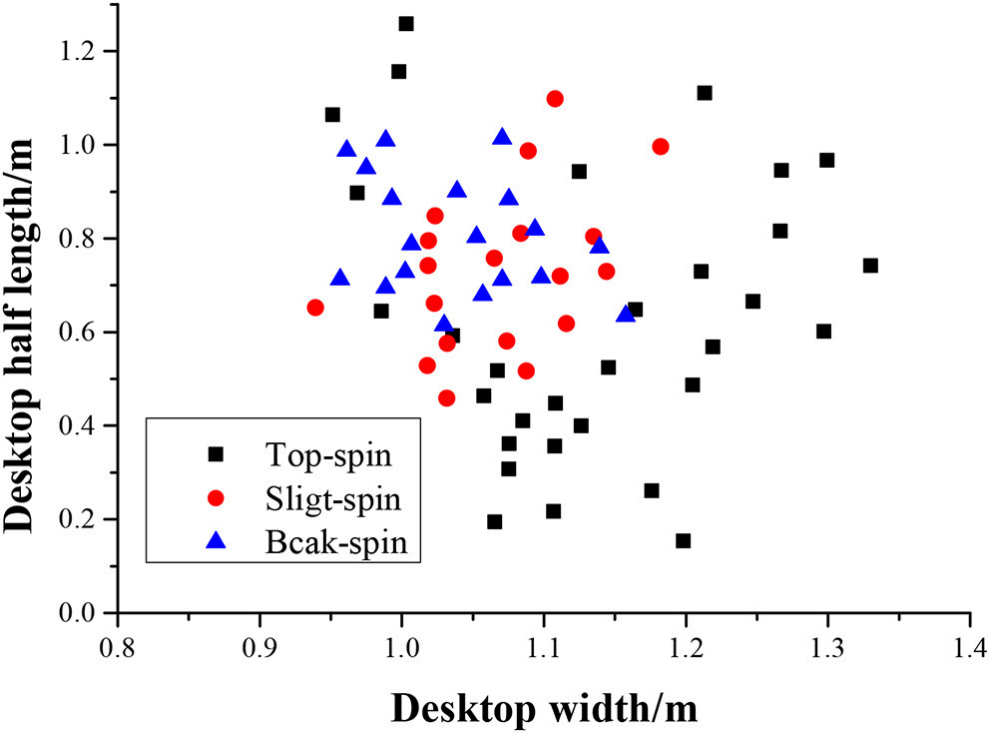
The detection distribution of the three difficulty services.

Three preset difficulty levels of slight-spin, top-spin, and back-spin can be achieved by adjusting the two parameters of the OUKEI automatic serve machine such as top-spin speed and back-spin speed. When the position of the automatic serve machine is fixed, the drop point distribution of the served ball can comprehensively reflect the variance of the curvature, flight time, and other indicators. The drop point distribution of the three difficulty services received by athletes is shown in Figure 10.

Figure 10 shows that the serving accuracy of slight-spin and back-spin is better than that of the top-spin. Hence, a lower serve speed has higher accuracy.

### Analysis Results of Motion Standardization of Athletes

**Figure 11:**
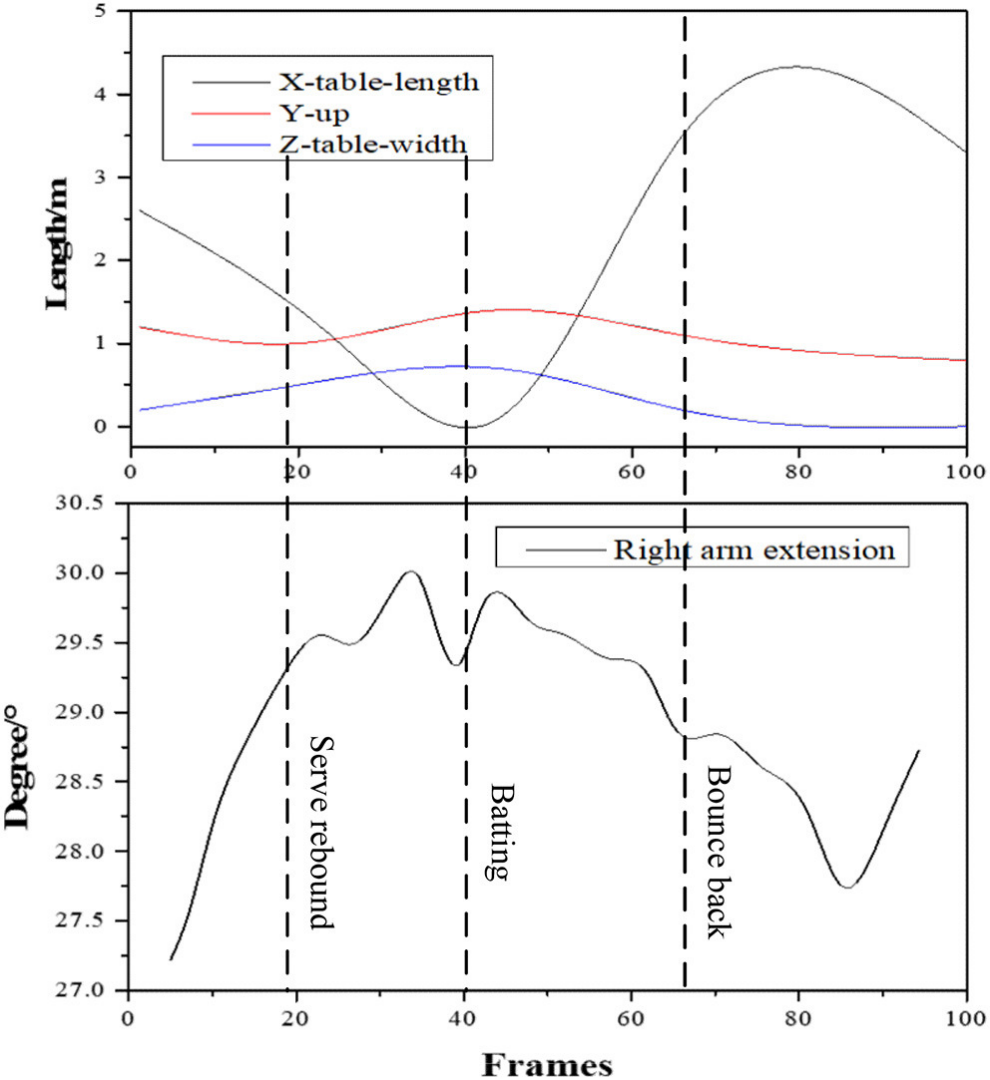
Comparison of the ball trajectory and right arm extension motion detection of athletes during the same round.

The physical motions of athletes are time-related to the hitting round; that is, the evaluation of the motion standardization refers to the evaluation of the motions corresponding to a particular hitting round. In the experiment, the analysis results of the ball trajectory and the right arm extension motion of athletes in the same round are shown in Figure 11.

The black curve, red curve, and blue curve in the trajectory diagram represent the position changes in the table tennis along the long side, vertical upward, and short side of the table, respectively. The lower side shows the change graph of the shoulder joint extension angle in the unit of the angle at the corresponding time. The absolute value of the extension angle increases, reflecting the forward swing of the arm of athletes. The maximum time of the extension angle corresponds to the hitting moment. Experimental results show that the level distribution of the three participants calculated by the system is consistent with the actual situation in terms of the quality of the ball returned and the standard of the motion, proving that the proposed KBCS and algorithm are useful in a small sample, laying a solid foundation for comprehensive and accurate sports evaluation and sports feedback in the future.

### Comparative Analysis of the Training Effect of Comparison Method and Traditional Method

**Figure 12:**
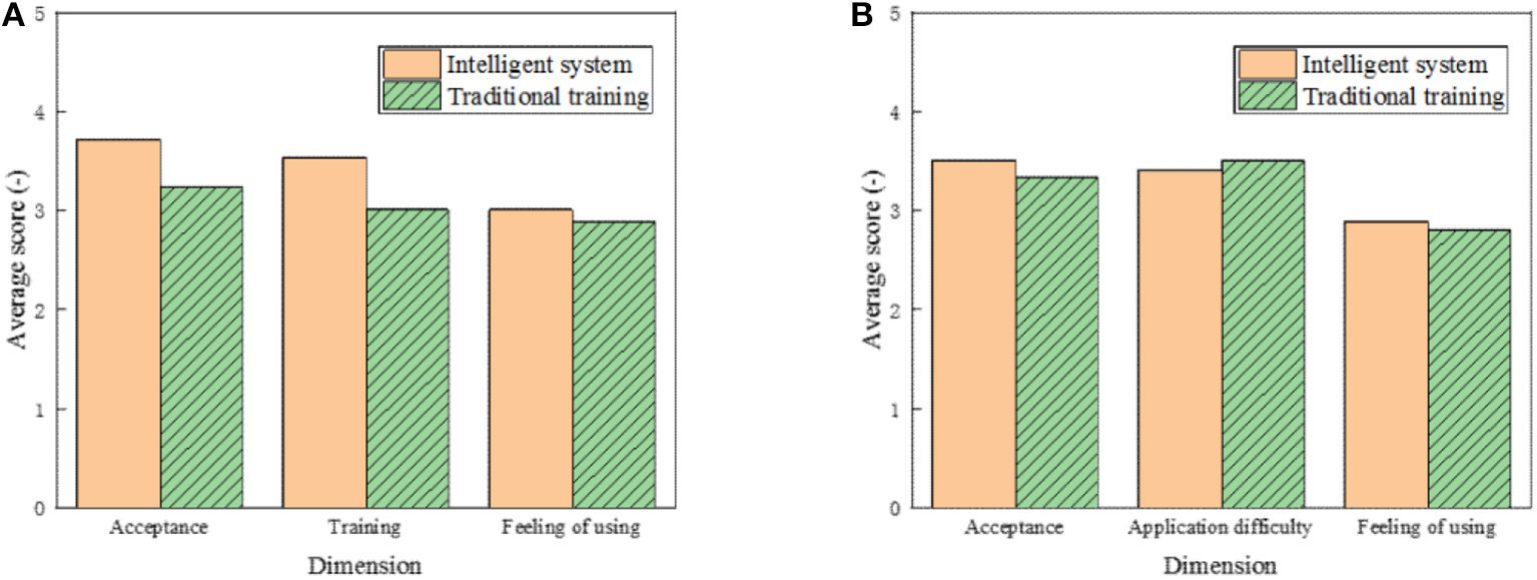
Comparative analysis of training effect of different systems.

To further explore the application effect of the proposed KBCS, the Questionnaire Survey (QS) method is selected, and 30 college athletes and six coaches are chosen as subjects. The 5-point Likert scoring method is used in the QS, in which, 1–5 points correspond to completely dissatisfied to completely satisfied, respectively. The higher the score is, the better the system effect is. College athletes are investigated from three aspects: acceptance, training enthusiasm, and user experience. Coaches are investigated from three dimensions: acceptance, application difficulty, and user experience. The specific results are shown in Figure 12. Figure 12A illustrates the QS results of college athletes, and Figure 12B demonstrates the QS results of coaches.

Figure 12A suggests that the acceptance of athletes of the proposed KBCS is the highest, 3.72 points, which is significantly higher than in the traditional method; the training enthusiasm of athletes for the system is 3.54 points, which is also significantly higher than in the traditional method; and the scores of user experience of the proposed intelligent system training method and the traditional training method are 3.01 and 2.89, respectively. Figure 12B indicates that acceptance scores of coaches of the proposed KBCS are 3.51, which is slightly lower than that of students, but still higher than that of traditional methods. However, in terms of application difficulty, the score of the proposed method is 3.41, which is lower than that of traditional methods. In terms of user experience, the scores of the two methods are 2.89 and 2.81, respectively, with a small gap.

## Discussion

Here, the specific training effect of the proposed table tennis training system is verified through a series of experiments. First, the three different serve modes are compared, namely, the slight-spin, top-spin, and back-spin serve. The proposed KBCS can mark the initial data in detail for serve modes with good system performance. Second, the system can also accurately measure the specific trajectory of the serve. The system can describe the abstract regulatory trajectory parametrically. Third, the proposed KBCS finds that the common serve errors are divided into two cases: The service is not over the net, or the landing point is not in the specified area. The specific performance of the two cases shows significant differences, in which the overall trajectory of the service without passing the net is relatively gentle, while the trajectory curve of the landing point not in the specified area is more tortuous, showing significant rebound.

In terms of the ball-receiving step, the problems in receiving the ball can be divided into three situations: missed receiving, receiving the ball out of the table, and receiving the ball touching the net. The specific content of these three situations can also be described parametrically through the proposed KBCS. In the actual experiment, the length of the X-direction in the error of ball-receiving shows a downward trend, while in the other two cases, the X-direction shows a rebound situation in the actual situation. At the same time, a standardized analysis for college athletes is established to compare the corresponding relationship between right arm extension and the trajectory of table tennis of college athletes. The results show that the standardized analysis results of the three college athletes are accurate, proving the effectiveness of the proposed KBCS.

The results of QS reveal that the overall application effect of the proposed KBCS is good. Compared with the traditional method, the proposed intelligent training method has higher acceptance and better user experience, fully improving the training enthusiasm of college athletes. The proposed method can display the standard training situation of athletes parametrically, scientifically understand the sports situation of college athletes, and effectively improve the effect of sports training. Therefore, the proposed KBCS can assist PE teaching and has a significant practical value. However, in the follow-up study, the utilization steps of the proposed KBCS should be further simplified, and its application scope should be expanded.

## Conclusions

A general coaching system based on hybrid motion capture technology, especially sports evaluation based on motion capture technology, is designed and proposed by analyzing and reviewing the current applications of artificial intelligence (AI) and HCI technologies in sports training and sports analysis. Then, this coaching system is applied to table tennis training and trajectory detection. The construction method of skill level model of athletes is designed from the perspective of interactive training. The optical motion capture system is adopted to capture the trajectory of table tennis and racket, and the inertial motion capture system is employed to restore the pose of the human body. Experimental results demonstrate that the human body pose correction method has good smoothness under the condition of the contact position constraint, which can further improve the accuracy of human inertial motion capture. Besides, the level distribution of the quality of the returning ball and the standard of the motion is consistent with the actual situation. The usability of the platform and the effectiveness of the sports evaluation method are verified preliminarily. According to the actual QS data, the application effect of the proposed method is significantly better than that of the traditional training method in acceptance, training enthusiasm, and user experience. The research results show that the proposed method has good feasibility. The results are scientific and effective and further can promote the development of parameterization, intelligence, and standardization of sports training, showing a significant practical value. However, due to the time and resource limitations, the number of sample subjects is small. In the follow-up study, the performance of the system will be further improved, so that the system is suitable for a large-scale data environment. Meanwhile, the system operation steps should be simplified to improve its reaction time and promote its applicability.

## Data Availability Statement

The raw data supporting the conclusions of this article will be made available by the authors, without undue reservation.

## Ethics Statement

The studies involving human participants were reviewed and approved by Zhoukou Normal University Ethics Committee. The patients/participants provided their written informed consent to participate in this study. Written informed consent was obtained from the individual(s) for the publication of any potentially identifiable images or data included in this article.

## Author Contributions

All authors listed have made a substantial, direct and intellectual contribution to the work, and approved it for publication.

## Conflict of Interest

The authors declare that the research was conducted in the absence of any commercial or financial relationships that could be construed as a potential conflict of interest.
